# Correction to: NFATc2 is an intrinsic regulator of melanoma dedifferentiation

**DOI:** 10.1038/s41388-019-0679-8

**Published:** 2019-01-28

**Authors:** V. Perotti, P. Baldassari, A. Molla, C. Vegetti, I. Bersani, A. Maurichi, M. Santinami, A. Anichini, R. Mortarini

**Affiliations:** 1 Human Tumors Immunobiology Unit, Department of Experimental Oncology and Molecular Medicine, Fondazione IRCCS Istituto Nazionale dei Tumori, Milan, Italy; 2 Melanoma and Sarcoma Unit, Department of Surgery, Fondazione IRCCS Istituto Nazionale dei Tumori, Milan, Italy

**Correction to: Oncogene;**

10.1038/onc.2015.355; Published online 21 Sept 2015

In Fig. 6e, the authors noticed that wrong blots for MITF, MART-1 expression/modulation, and for β-actin were presented, due to the similarity with experiments shown in Figure 5c. Correct MITF, MART-1, and β-actin blots were added to the revised Fig. 6 shown in the associated Correction. The meaning of the results shown in Fig. 6e, as well as the conclusions of this paper were not affected, and the authors regret for this error. These errors have not been fixed in the original Article.

**Fig. 6:**
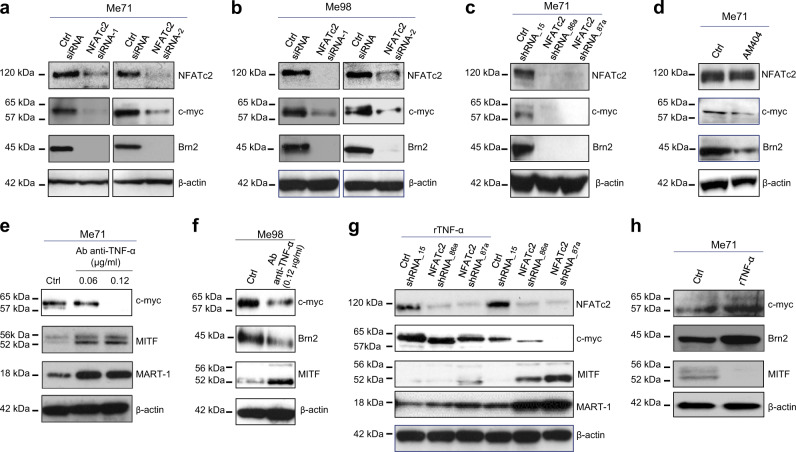
▓

